# Medication-Related Burden among Patients with Chronic Disease Conditions: Perspectives of Patients Attending Non-Communicable Disease Clinics in a Primary Healthcare Setting in Qatar

**DOI:** 10.3390/pharmacy6030085

**Published:** 2018-08-13

**Authors:** Amani Zidan, Ahmed Awaisu, Maguy Saffouh El-Hajj, Samya Ahmad Al-Abdulla, Dianne Candy Rose Figueroa, Nadir Kheir

**Affiliations:** 1 College of Pharmacy, Qatar University, Doha 2713, Qatar; az1405317@qu.edu.qa (A.Z.); aawaisu@qu.edu.qa (A.A.); maguyh@qu.edu.qa (M.S.E-.H.); 2 Primary Health Care Corporation, Doha 2713, Qatar; saalabdulla@phcc.gov.qa (S.A.A-.A.); dfigueroa@phcc.gov.qa (D.C.R.F.); 3 School of Pharmacy, Faculty of Medical and Health Sciences, University of Auckland, Auckland 1010, New Zealand

**Keywords:** medication-related burden, questionnaire, chronic disease conditions, adherence

## Abstract

This study aimed to assess perceived medication-related burden among patients with multiple non-communicable diseases (NCDs) and to investigate the association between perceived burden and adherence to medication therapy. Using a cross-sectional study in three primary care clinics in Qatar, medication-related burden was measured using the Living with Medicines Questionnaire (LMQ) among adults with diabetes, with or without other comorbidities. Adherence was measured using the Adherence to Refills and Medications Scale (ARMS). Two hundred and ninety-three eligible patients participated in the study. The majority of them reported experiencing minimal (66.8%) to moderate (24.1%) medication-related burden. There was a significant positive correlation between the scores of the LMQ (medication-related burden) and ARMS (medication adherence), rs (253) = 0.317, *p* < 0.0005.

## 1. Introduction

Non-communicable diseases (NCDs) are associated with an increasing prevalence of morbidity and mortality globally [1]. Despite concerted global efforts aimed at reducing the burden of these diseases [2,3,4], the main focus of healthcare systems and clinical practice guidelines in general is to achieve and maintain clinical therapeutic goals for single conditions when managing chronic illnesses [5,6,7]. This type of fragmented care approach would lead to diminished quality of life among people with multimorbidities [7].

Medications are commonly used for the long-term management of chronic diseases [8]. The benefits of medications in preventing or slowing the progression of the adverse consequences of NCDs, including premature deaths, as well as managing the associated symptoms, are indisputable [8]. Polypharmacy, defined as the use of multiple medications [9], could be perceived as an unavoidable consequence of the advancement in the treatment strategies of today’s aging population [9,10,11]. Paradoxically, polypharmacy has also been associated with increased morbidity and mortality, hospitalizations, and demand for nursing home care [12,13].

The traditional focus of clinical practice guidelines on individual diseases, the increasing coexistence of multiple comorbid conditions, and the lack of structured strategies to manage the problems that are associated with the consequences of treatments means that patients with multiple NCDs have to deal with extremely complex instructions and tasks that are associated with medications for the rest of their lives [14]. Coping with the adverse consequences of medications and having to tailor their life activities according to the demands of therapeutic regimens places an extra burden on patients [6,15,16]. Thus, the experience of using medications and health care, especially for long durations, as a potentially critical threat to the success of treatment regimens, is worth investigating [17,18].

The concept of “medication-related burden” or treatment burden has been well-described in the literature as the overall workload that is imposed on patients resulting from utilizing health care, leading to multiple negative effects in their lives [15,17,19,20,21]. Studies have also highlighted the association between this burden and adherence to medication therapy [6,15,21,22]. Although the concept of treatment burden is increasingly attracting attention from various research groups, studies that mainly focus on the association between the treatment burden and adherence to therapy are scarce, mainly use qualitative methodology, and focus on specific diseases [23,24].

Qatar’s National Health Strategy and its updates declared the establishment of a world-class healthcare system aiming to provide a comprehensive primary care model that puts the patient at the center of care [25,26,27]. Through the NCD clinics that have been established in primary health centers that provide care for all patients with chronic diseases in Qatar, the Primary Health Care Corporation (PHCC) guarantees primary care services emulating the recommended transition in health care globally, and also has plans and strategies that are aimed at reducing the mortality and burden that is related to the NCDs [25,26].

The assessment of the medication-related burden from the patient’s perspective is an important endeavor to appraise the National Health Strategies of any barriers that may hinder the optimum use of health services at the primary health care level. Moreover, previous studies have qualitatively reported the association between perceived medication-related burden and patients’ well-being, as well as adherence to therapy. To our knowledge, this association has not yet been quantitatively measured among patients with NCDs. This study aims to assess the burden that is resulting from the treatment of chronic NCD conditions in Qatar and its impact on medication adherence.

## 2. Materials and Methods

This cross-sectional study was conducted among patients with chronic NCDs in Qatar to measure perceived medication-related burden and its association with medication adherence. Ethical approval to conduct the study was obtained from the Research Section (Clinical Affairs) of the PHCC (approval no. RC Ref. PHCC/RC/15/10/015).

Primary health care services are provided by the PHCC to patients with NCD living in Qatar through 23 health centers that host NCD clinics [26,27]. For this study, three centers providing such services were selected. The selection of the clinics was based on the ethical approval conditions, the approximate similarity in the demographic distribution of the patients’ visits across the health centers, and was based on the fact that all of the three centers had NCD clinics [26].

Although the perceived medication-related burden is expected to be associated with seeking treatment for any chronic illness [21], patients in this study were recruited if they had diabetes mellitus (DM), with or without comorbidities (other NCDs). DM can be considered as a representative example of patients living with chronic conditions and is an NCD of high priority in Qatar due to its high prevalence [26]. Patients were eligible for enrollment in the study if they were at least 18 years of age, had been diagnosed with diabetes for at least 6 months prior to the study (with or without other comorbidities), and were able to communicate in English and/or Arabic. As 13.5% of the population in Qatar has diabetes [28], and the medication-related burden was assumed to exist with all of them, the sample size was estimated using the following equation [29]:

Sample size = (Z_1-a/2_) × 2 × P × (1 − P)/d^2^

where Z_1-a/2_ is standard normal variate, which is 1.96 at a 5% level of confidence; P is the expected proportion in the population (*p* = 0.135); and assuming an absolute error (d) of 0.05. This number was increased by 30% to account for missing data. Hence, a total of 234 patients was the target for this study.

The primary outcome measure was the self-reported medication-related burden (including LMQ score and the VAS score). Self-reported adherence was assessed as a secondary outcome measure.

### 2.1. Study Instruments

Below is a description of the instruments that were used in the study:

(1) The Living with Medicines Questionnaire (LMQ) [30,31]: This is a 41-item questionnaire with which respondents are required to indicate their level of agreement using a five-point Likert-type scale (from strongly agree to strongly disagree). In addition, a free text (open-ended) question gives the respondent the opportunity to add any other relevant issues that are not covered in the questionnaire. The tool was comprised of eight domains: Relationships with health professionals, Practicalities, Information, Efficacy, Side effects, Attitudes, Impact, and Control. The overall LMQ score was the sum of the scores of all of the 41 items in the questionnaire, and ranged from 41 to 205, with higher scores indicating greater burden. The questionnaire also contained a visual analogue scale (VAS), through which respondents provided a global assessment of the overall burden they experienced (0 to 10 points, with higher scores representing greater perceived burden). This tool was validated in English [30] and was adapted into the Arabic context using best practices [31]. Both the Arabic and the English versions were used in this research, as applicable.
(2) The Adherence to Refills and Medication Scale (ARMS) [32]: This is a 12-item questionnaire that had also been validated in English and translated into the Arabic context by the research team in coordination with the original developers of the tool. The ARMS score was the sum of the scores of the 12 items on the scale, and ranged from 12 to 48, with higher scores indicating worse adherence. The ARMS was developed to measure adherence to drug therapy and was validated among patients that had been prescribed long-term therapy for coronary heart diseases [32]. The scale demonstrated a high internal consistency reliability (Cronbach’s α = 0.814) and a significant correlation with the Morisky Adherence Scale (Spearman’s rho = −0.651, *p* < 0.01) [32]. Both the Arabic and the English versions were used in this research, as appropriate.

### 2.2. Data Analysis

Descriptions and comparisons using frequencies and percentages were used to describe all of the variables in the study and to express the perceived medication-related burden among the participants. Inferential statistics (univariate analysis) were also used to determine and compare the medication-related burden scores across the different demographic and clinical characteristics. In order to demonstrate the relationship between the perceived medication-related burden and adherence to medication therapy, correlation analysis was applied [33]. As reported in the literature, perceived medication-related burden was assumed to be associated with medication adherence [21,22]. Given the cyclic nature of perceived burden [21], the direction of this association was not hypothesized in this study.

## 3. Results

Of the 500 eligible patients approached, 307 consented to participate in the study. After excluding forms with incomplete or invalid data (i.e., the participant only responded to one questionnaire or responded with neutral response to all of the items of the LMQ), a total of 293 participants were included in the analysis. Among them, 86.3% provided complete responses to all of the items in the LMQ.

Table 1 and Table 2 contain the sociodemographic, clinical, and other characteristics of the study sample. Most of the participants were young to middle aged adults (78.4%), male (71%), non-Qataris (non-Qatari Arabs 41.6%), married (94.9%), educated (54.3% with a university degree or higher), and employed (70.4%). The majority of the participants (66.6%) reported that they were not following any lifestyle changes that had been recommended by their healthcare providers. Smoking history (cigarette and/or shisha) revealed that the majority of the studied cohort had never been a smoker.

The median (IQR) duration of the DM diagnosis was 8.0 (8.0) years, with the majority (66.6%) diagnosed from 6 months to 10 years ago. Most of the participants (90.1%) had comorbidities, with 77.1% having up to three comorbidities. The most commonly reported comorbidities were hypertension (55.3%), dyslipidemia (55.3%), and obesity (48.1%). Participants were prescribed with a median (IQR) of 5.0 (3.0) medications and 6.0 (3.0) daily doses. In addition, approximately 29% of the participants were prescribed more than five medications. The diabetes control status of the patients was determined using the most recently available HbA1c value in the medical records. The median (IQR) HbA1c value was 7.80% (2.3) and 66.2% of the participants had uncontrolled DM (HbA1c greater than 7%). In addition, the median (IQR) Body Mass Index (BMI) of the study participants was 29.98 (6.68) kg/m^2^.

The perceived medication-related burden was measured among the participants using the LMQ. The median (IQR) LMQ score and VAS score were 95.00 [22] (possible range: 41 to 205) and 3.00 (4) (possible range: 0 to 10), respectively. These findings showed that the majority of the participants experienced minimal (66.8%) to moderate (24.1%) degrees of burden (Table 3).

Adherence was measured using ARMS, and the results showed that 84% of the participants were non-adherent to their prescribed medications (Table 4).

Mann-Whitney *U* and Kruskal-Wallis tests were used to determine the influence of the sociodemographic and clinical characteristics of the participants on the perceived medication burden. The median LMQ score for Qatari was significantly higher, representing worse medication-related burden than that for non-Qatari, (*p* = 0.011). The participants who had spouses showed significantly lower LMQ scores than the participants who did not (*p* = 0.002). There were statistically significant differences for the median LMQ scores between the employed and non-employed participants (*p* = 0.044). Furthermore, the participants who had been diagnosed with DM for more than 10 years had a statistically significantly higher median LMQ score than that of the participants who had the diagnosis for less than 10 years (*p* = 0.007).

According to the VAS scores representing global burden, the participants with uncontrolled DM reported significantly higher global burden than the participants with controlled DM (*p* = 0.018). The median VAS score for the participants who had been diagnosed with DM for more than 10 years was significantly higher than that for the participants who had been diagnosed with DM for less than 10 years (*p* = 0.043).

A Spearman’s rank-order correlation test was used to assess the relationship between the perceived medication-related burden and adherence to prescribed medications among the study population. There was a moderate positive correlation between LMQ score and ARMS score, *r_s_* (251) = 0.317, *p* < 0.0005. This correlation implies that the higher the medication-related burden is, the lower the medication adherence level is (given that the higher ARMS score translates into lower adherence and that the higher LMQ score translates into greater burden). There was also a moderate positive correlation between the VAS scores and the ARMS score, *r_s_* (284) = 0.325, *p* < 0.0005. This also indicates that the more the perceived medication burden is, the lower the level of medication adherence is.

## 4. Discussion

To our knowledge, this study was the first to measure medication-related burden from the perspective of patients living with chronic diseases, attending NCD clinics at PHCC in Qatar. As the majority of the patients visiting NCD were suffering from diabetes, we deliberately investigated medication-related burden among patients with diabetes as the main NCD disease focus. The LMQ was used to measure the aspects of medication-related burden that were experienced by the NCD patients. Although almost all of the participants that were interviewed found this measure extremely relevant, the majority of them commented on the length of it. Our cohort of patients resembled the population in Qatar [34], with the majority of them being males and from different nationalities. As expected, and similar to previous studies [35,36,37], most of our participants suffered several comorbidities, had been prescribed several medications, were non-adherent to their therapy, had uncontrolled diabetes, and adopted a sedentary lifestyle.

As the interest in conceptualizing and measuring medication-related burden is relatively new, there is currently only a few studies to compare our results to. To our knowledge, this is one of the first studies to assess medication-related burden among patients with NCDs from the perspective of the patients as an independent measure from the disease or medication context [36]. Our study indicated that a considerable proportion of the participants (90%) were suffering from varying degrees of burden that was related to their medication and overall treatment. As expected, this burden was minimal to moderate, given the high quality of services that was provided to the NCD patients in Qatar at minimal cost and in one clinical setting. The results of the current study can best be compared to the results of a recent study that was conducted in Australia, which assessed the overall treatment burden among patients with chronic conditions [36]. Although this study by Sav. et al. used a different tool (The Treatment Burden Questionnaire; TBQ), the main focus of their measurement was still close to that of the current study. They also found that, independently from the ailment itself, treatment burden affected a considerable proportion of the patients with chronic diseases. Similar to our study, they further highlighted the effects of patients’ characteristics on the perceived burden [36].

Additionally, participants of Qatari nationality, female gender, who were unmarried, unemployed, had been diagnosed with DM for more than 10 years, had uncontrolled DM, and had been prescribed medication types other than tablets or capsules, demonstrated significantly higher scores of medication burden. Cultural differences between Qatari nationals and non-Qatari residents could translate into different levels of perceived burden resulting from medication therapy. As indicated in other studies, females tended to show higher levels of burden than males [20,38]. These findings also indicate that having someone to provide support and having a job could reduce the burden perceived by the patient. Our results also highlight the impact of the controlled status of the chronic condition on living with less burden. As expected, living longer with the disease, or being prescribed with any other dosage form other than pills, could translate into more discomfort with the treatment and its consequences. This also could be explained by the progressive nature of diabetes, which gets worse with time, requiring more activities of utilizing health care, and translates into perceiving greater burden.

This study also found significant positive association between the scores of medication burden and self-reported medication adherence, which further supports previous qualitative studies suggesting a lack of adherence among patients who experience medication burden [11,20]. In a study that used the TBQ among patients with chronic conditions from many English-speaking countries, Tran et al. found higher perceived burden among patients with lower levels of adherence [16].

This study has several limitations that warrant mentioning to benefit future research. First, since it was a cross-sectional study, it implies that we were unable to capture all of the factors that might have affected medication-related burden over time. A longitudinal study would be more capable of investigating the effect of those factors on perceived burden over time. Second, while self-reported adherence is still considered to be the most feasible, user-friendly, and simple means to measure medication adherence [39], a combination of subjective and objective methods of measuring adherence is recommended [40]. Third, this study was limited to patients who were able to communicate in English or Arabic. Hence, the results cannot be generalized to people coming from different cultures, constituting a considerable proportion of the population in Qatar. In fact, perceived burden could be affected by factors that are related to the differences in beliefs about medications [21], and such differences can also be related to culture. Future studies investigating these factors are recommended. Fourth, although our sample demographic information resembles those of the population in Qatar, it may not be representative of it due to the limitation of the sampling technique. Finally, although we have attempted to investigate the association between adherence and medication-related burden, the results of this investigation could be considered preliminary, and future studies (qualitative and quantitative) that focus on highlighting the predictors of medication-related burden are needed.

## 5. Conclusions

A considerable proportion of patients experienced medication-related burden, which could be affected by many factors including adherence to drug therapy, the duration of the chronic disease diagnosis, control of the disease, being employed, or receiving support from family. Our study’s findings suggest that healthcare professionals should be aware of the impact of treatment plans on the lives of patients with chronic diseases. In addition, factors affecting medication-related burden should be taken into consideration when designing tailored interventions to reduce this burden.

## Figures and Tables

**Table 1:** Sociodemographic characteristics of the participants (N = 293).

Variable	Frequency (%)
Age (years)	
Up to 65	256 (87.4)
Above 65	37 (12.6)
Gender	
Male	208 (71.0)
Female	85 (29.0)
Country of origin/ethnicity	
Qatar	41 (14.0)
Arab countries (excluding Qatar) *	122 (41.6)
Indian subcontinent **	107 (36.5)
Philippines	14 (4.8)
Others ***	9 (3.1)
Education Level	
Less than primary school	3 (1.0)
Primary or middle school	47 (16.0)
Secondary school	52 (17.7)
Technical college	32 (10.9)
University degree	145 (49.5)
Postgraduate degree	14 (4.8)
Marital status	
Married	278 (94.9)
Single	7 (2.4)
Divorcee	5 (1.7)
Widowed	3 (1.0)
Lifestyle changes	
None	184 (62.8)
Exercise	103 (35.2)
Exercise & healthy diet	6 (2)
Cigarette smoking	
Current smoker	32 (10.9)
Former smoker	42 (14.3)
Never been a smoker	219 (74.7)
Shisha smoking	
Current daily smoker	8 (2.7)
Current social smoker	11 (3.8)
Former smoker	16 (5.5)
Never been a smoker	258 (88.1)
Employment	
Employed	205 (70.4) ****
Unemployed	68 (23.4) ****
Retired	17 (5.8) ****
Full-time student	1 (0.3) ****

* Arabs countries include: Egypt, Jordan, Lebanon, Palestine, Sudan, Syria, Yemen, Iraq, Tunisia, and Morocco. ** Indian subcontinent include: India, Pakistan, Sri Lanka, and Bangladesh. *** Others include: Eretria, Hungary, Iran, Germany, Canada, Kenya, Brazil, and Britain. **** Percentage totals may not be 100% due to some missing responses.

**Table 2:** Clinical Characteristics of the Study Participants (N = 293).

Variable	Median (IQR)	Frequency (%)
Duration of the DM diagnosis	8.0 (8.0)	
6 months to 10 years		167 (66.5) *
More than 10 years		84 (33.5) *
Presence of co-morbidities		264 (90.1)
Number of co-morbidities		
One		93 (31.7)
Two		104 (35.5)
Three or more		67 (22.9)
Hypertension		162 (55.3)
Dyslipidemia		162 (55.3)
Vitamin D deficiency		18 (6.1)
Thyroid dysfunction		10 (3.4)
Obesity		141 (48.1)
Asthma		6 (2)
Others *		19 (6.5)
Number of prescribed medications	5.0 (3.0)	
Up to 5 medications daily		208 (71)
More than 5 medications daily		85 (29)
Medication type		
Tablet/capsules		211 (72)
Any other type		82 (28)
Help with medicines		88 (30.4) *
HbA1c	7.80% (2.3)	
DM control status		
Controlled **		85 (29) *
Uncontrolled		194 (66.2) *
BMI *** (Kg/m^2^)	29.98 (6.68)	

* Percentage totals may not be 100% due to some missing responses. ** (HbA1c ≤ 7%). *** Body Mass Index.

**Table 3:** Perceived Medication-Related Burden Measured Using the Living with Medicines Questionnaire (LMQ) in Patients Attending non-communicable disease (NCD) Clinics in Qatar (N = 293).

Variable	Range	Mean (SD)	Median (IQR)	Frequency (%)
LMQ overall score *	(41–205)	97.5 (18.6)	95.0 (22)	
No burden at all	(41–73)			18 (7.1)
Minimal burden	(74–106)			169 (66.8)
Moderate degree of burden	(107–139)			61 (24.1)
High burden	(140–172)			5 (2)
Extremely high burden	(173–205)			-
Theme 1: Relationships with healthcare professionals about medicines	(5–25)	9.74 (3.12)	9.0 (4.0)	
Theme 2: Practical difficulties	(7–35)	15.19 (4.0)	15 (5.0)	
Theme 3: Cost-related burden	(3–15)	6.75 (2.80)	6.0 (4.0)	
Theme 4: Side effects of prescribed medications	(4–20)	9.65 (3.72)	8.0 (5.0)	
Theme 5: Effectiveness of medicines	(6–30)	11.36 (2.9)	12.0 (3.0)	
Theme 6: Attitudes/concerns about medicine use	(7–35)	20.35 (5.3)	20.0 (9.0)	
Theme 7: Impact/Interference to day-to-day life	(6–30)	14.31 (4.4)	13.0 (6.0)	
Theme 8: Control/ Autonomy to vary regimen	(3–15)	10.17 (2.6)	10.0 (4.0)	
VAS: global burden	(0–10)	3.17 (2.5)	3.0 (4)	

* Total of LMQ with complete responses is 253 due to some missing responses.

**Table 4:** Self-Reported Adherence of Patients with Chronic Conditions Attending NCD Clinics in Qatar Measured By Adherence to Refill and Medications Scale (ARMS) (N = 293).

Variable	Mean (SD)	Median (IQR)	Frequency (%)
ARMS overall score	17.4 (4.8)	16.0 (7)	
Adherent			47 (16)
Non-adherent			246 (84)
